# Evaluation of Three Chitin Metal Silicate Co-Precipitates as a Potential Multifunctional Single Excipient in Tablet Formulations

**DOI:** 10.3390/md8051699

**Published:** 2010-05-25

**Authors:** Rana Al-Shaikh Hamid, Faisal Al-Akayleh, Mohammad Shubair, Iyad Rashid, Mayyas Al Remawi, Adnan Badwan

**Affiliations:** 1 Department of Pharmaceutics and Pharmaceutical Technology, Faculty of Pharmacy and Pharmaceutical Sciences, Petra University, Jordan; E-Mails: rana_ali19742000@yahoo.com (R.A.-S.H.); faisal971@yahoo.com (F.A.-A.); shubms@hotmail.com (M.S.); 2 Suwagh Company for Drug Delivery Systems Subsidiary of the Jordanian Pharmaceutical Manufacturing Co. (PLC), Naor, Jordan; E-Mails: irashid@jpm.com.jo (I.R.); mayyas_nj@yahoo.com (M.A.R.)

**Keywords:** chitin metal silicate co-precipitates, direct compression, wet granulation, multifunctional single tablet excipient

## Abstract

The performance of the novel chitin metal silicate (CMS) co-precipitates as a single multifunctional excipient in tablet formulation using direct compression and wet granulation methods is evaluated. The neutral, acidic, and basic drugs Spironolactone (SPL), ibuprofen (IBU) and metronidazole (MET), respectively, were used as model drugs. Commercial Aldactone^®^, Fleximex^®^ and Dumazole^®^ tablets containing SPL, IBU and MET, respectively, and tablets made using Avicel^®^ 200, were used in the study for comparison purposes. Tablets of acceptable crushing strength (>40 N) were obtained using CMS. The friability values for all tablets were well below the maximum 1% USP tolerance limit. CMS produced superdisintegrating tablets (disintegration time < 1 min) with the three model drugs. Regarding the dissolution rate, the sequence was as follow: CMS > Fleximex^®^ > Avicel^®^ 200, CMS > Avicel^®^ 200 > Dumazole^®^ and Aldactone^®^ > Avicel^®^ 200 > CMS for IBU, MET and SPL, respectively. Compressional properties of formulations were analyzed using density measurements and the compression Kawakita equation as assessment parameters. On the basis of DSC results, CMS co precipitates were found to be compatible with the tested drugs. Conclusively, the CMS co-precipitates have the potential to be used as filler, binder, and superdisintegrant, all-in-one, in the design of tablets by the direct compression as well as wet granulation methods.

## 1. Introduction

Excipients are known to have a defined function when incorporated in pharmaceutical dosage forms. These are added to modulate manufacturing and delivery of drug substances. The better the excipient can fulfill these functions, the more ideal that excipient is [1].

In solid dosage forms, particularly immediate release tablets and capsules, excipients are added to facilitate processing because they are used to bulk the product, lubricate the powder, ease the flow and facilitate compression of the powder bed [2]. Stability and solubility of the drug substance can be improved by including suitable excipients. This improvement enhances the bioavailability [3]. However, the introduction of an added excipient to the formulation brings with it new challenges concerning the drug-excipient interaction [4]. Therefore, manufacturers of solid dosage forms are always trying to reduce the number of excipients in their products. In addition, the introduction of new high speed tablet machinery requires excipients with good compressibility, compactablity**,** flow properties and even short dwell times. These requirements for such excipient specifications have led to most available ones falling short in fulfilling their role.

Excipients can be modified by manufacturing process to produce new grades of the present excipients, for example the modification of starch to become soluble or spray dried lactose to assist its followability [5]. Common excipients are modified by coprocessing with other excipients in order to offer excipients with added advantages. For example: Microcrystalline cellulose (MCC) was coprocessed with (silicone dioxide) SiO_2_ to obtain a high compatibility and high intrinsic flow with enhanced lubricant activity, yielding a better tablet hardness especially when wet granulation was used [6]. Coprocessing of lactose or calcium carbonate with MCC produced excipient suitable for direct compression [7,8].

Still there is a need for new excipients, which has led to some research groups suggesting the usage of chitosan [9]. Recently, chitosan was evaluated for its function as an excipient due to its ability to act as a fast disintegrating agent. Its flowability was improved by coprocessing with SiO_2_ [10]. Later, this co-processed chitosan was found to suffer from chemical activity with many drug substances due to the presence of free NH_2_ and SiO_2_ groups. This resulted in the development of a novel excipient based on chitin coprocessed with silica. Such modification overcomes the chemical reactivity in chitosan SiO_2_ mixture [11].

Recently, Rashid *et al.* [12] prepared and characterized three co precipitates of metal silicates (*i.e.*, calcium, magnesium, and aluminum) onto chitin particles, which can be compressed into rapid disintegrating compacts. The different metal silicates co-precipitated on the chitin particles provide different excipient pH, for example, the pH of chitin-Al silicate, chitin-Mg silicate, and chitin-Ca silicate are ranging between 3–6, 9.5–10, and 10–11 respectively. Consequently, it becomes necessary to test the suitability of these excipients to be used as a single component mixed with some active drug substances and to study their influence on the tablet’s properties and preparation methods.

In the present work, the use of co-precipitates of chitin Ca, Mg, and Al silicates as a single multifunctional excipient in tablet preparation of problematic drugs: ibuprofen (IBU), metronidazole (MET) and spironolactone (SPL) is evaluated.

## 2. Results and Discussion

### 2.1. Preparation and Characterization of Metal Silicate and Their Compacts

Ca, -Mg, and -Al ions are capable of forming complexes with silicate ions, leading to the formation of insoluble metal silicates according to the following equations, respectively:

(1) $${\text{CaCl}}_{2}+{\text{Na}}_{2}{\text{SiO}}_{3}\to {\text{CaSiO}}_{3}+2\text{NaCl}$$

(2) $${\text{MgCl}}_{2}+{\text{Na}}_{2}{\text{SiO}}_{3}\to {\text{MgSiO}}_{3}+2\text{NaCl}$$

(3) $$2{\text{AlCl}}_{3}+3{\text{Na}}_{2}{\text{SiO}}_{3}\to {\text{Al}}_{2}{\left({\text{SiO}}_{3}\right)}_{3}+6\text{NaCl}$$

These insoluble metal silicates were deposited on a chitin surface as reported earlier [12]. The pH of the media of the co-precipitation of calcium, magnesium, and aluminum silicates on chitin particles was measured as 10, 9, and 4, respectively. This highlights the basic nature of calcium and magnesium cations and the acidic nature of aluminum cations, which can be utilized in improving the dissolution of acidic and basic drugs. In a previous study, the resulted co-precipitate was found to be an amorphous powder [12].

### 2.2. Drug Excipient Compatibility

**Figure 1:**
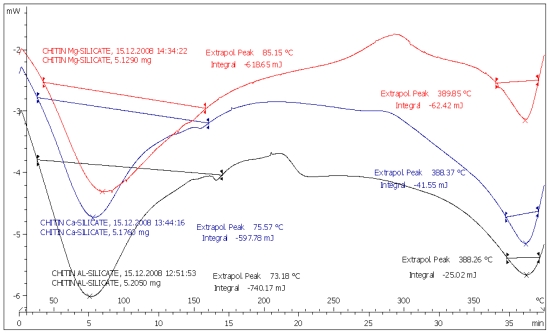
DSC of chitin Ca silicate (blue), chitin Mg silicate (red) and chitin Al silicate (black).

**Figure 2:**
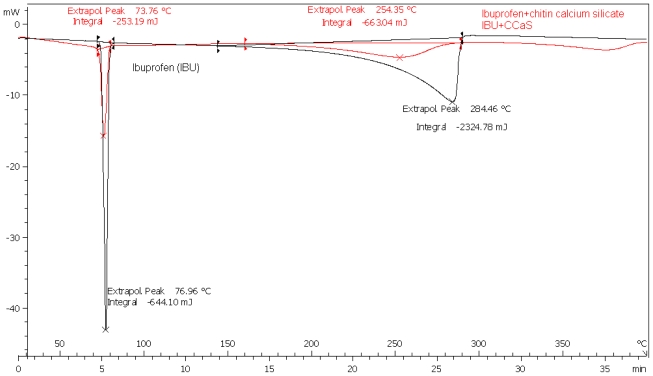
Comparison between ibuprofen alone (IBU) and ibuprofen with chitin calcium silicate (IBU + CCaS).

Differential scanning calorimetry (DSC) was used as a technique to measure the compatibility of the drugs with chitin-metal silicate (CMS) by measuring the physical transformation of the drugs when they undergo phase transitions due to endothermic or exothermic heat flows. Examples of such thermograms are presented in Figures 1 and 2.

All the CMS(s) showed broad spectrum endothermic peaks at 30–120 °C (Figure 1). Many authors [13] related the presence of such broad spectrum peak in this region to water evaporation of polymeric materials.

Sharp endothermic peaks due to melting at 76, 160 and 210 °C were recorded for IBU, MET and SPL, respectively. Endothermic peaks shown above 250 °C are due to the decomposition of chitin itself. The melting endotherms of the physical mixture of the drugs with CMS were well preserved with slight changes in terms of broadening or shifting towards lower temperatures (Figures for MET and SPL are not shown). It has been reported that the quantity of material used, especially in drug-excipient mixtures, affects the peak shape and enthalpy. Thus, these changes in the melting endotherm could be due to the mixing process, which lowers the purity of each component in the mixture, and may not necessarily indicate potential chemical incompatibility [14]. However, as the physical interaction may affect the crystalline structure, this may be correlated to the observed lowering in the melting endotherms. Such results indicate no chemical interaction is detectable.

### 2.3. Compression Studies Analysis

The compression behavior of the three model drugs, CMgS, and mixture of CMgS with the drugs were analyzed using the Heckel equation (Equation 4).

Powders of MET and SPL failed to form solid compacts at the pressures applied (2.7–44.2 MPa), while IBU was compressed easily over this pressure range. However, it was not possible to obtain Heckel plots since these powders form non porous solid that formed a low volume tablet.

Heckel plots (not shown) of CMgS, CMgS with IBU, MET, or SPL showed pronounced initial curvature followed by a linear portion. This is an indicator of intensive particle rearrangements and fragmentation followed by plastic deformation. All tested formulas showed similar Heckel plots, which mean that the addition of drugs did not affect the shape of the Heckel plot. Different parameters were obtained from Heckel plots and are presented in Table 1. The *P_y_* values, which represent the yield pressure (*i.e.*, the least pressure at which particle plastic deformation is initiated), decreased from 153.9 MPa; for the single excipient, *i.e.*, chitin Mg silicate (CMgS), to 82, 86.3, and 103.1 MPa for the mixtures of the same excipient with IBU, MET and SPL drugs, respectively (calculated *p* < 0.05 of the yield pressure values between that of the single excipient and that of the excipient with the incorporated drugs). This implies that, the onset of plastic deformation of CMgS occurred at lower pressure in the presence of drugs [15]. This implies that CMgS can be useful in high-speed tablet machines with short dwell time, where faster onset of plastic deformation should be useful in avoiding problems of lamination and capping [16].

The *D_0_* value, which represents the degree of initial packing in the die as a result of die filling for CMgS, increased in the presence of drugs and were in the order: CMgS with IBU > CMgS with MET > CMgS with SPL > CMgS. This shows that the CMgS/drug mixtures exhibit higher degree of packing in the dies as a result of die filling than CMgS powder alone.

The value of *D_B_* represents the phase of rearrangement of the particles in the early stages of compression. The *D_B_* value of CMgS powder alone was 0.36 and increased to 0.44, 0.48, 0.49 for CMgS with IBU, CMgS with MET, and CMgS with SPL, respectively. This increase indicates that the fragmentation of the powder mixture is more pronounced than the CMgS alone. The value of *D_A_*_,_ which represents the total degree of packing achieved at zero and low pressure (*i.e.*, *D_0_* + *D_B_*), increased for CMgS in the presence of drugs.

**Figure 3:**
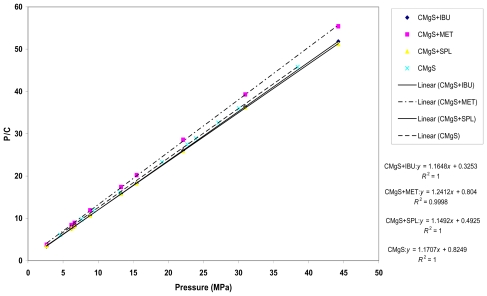
Kawakita plots of chitin Mg silicate alone (CMgS) and with ibuprofen (IBU), metronidazole (MET), and spironolactone (SPL).

Figure 3 showed Kawakita plots for CMgS alone and CMgS with IBU, MET and SPL, respectively. A linear relationship between *P/C* and *P* was obtained at all applied compression pressures with a correlation coefficient ≥ 0.99. Values of “*1/a*” and “*1/ab*” were obtained from the slope and intercept of the plots, respectively, and are summarized in Table 2.

From the table, the value of “*a*” parameter, which represents the compressibility (total volume reduction) of the powder before compression, for CMgS alone was not significantly different (*p* > 0.05) from the “*a*” values of CMgS with the drugs. This means that the addition of a drug to the excipient did not significantly alter the measured compressibility of the powder.

The value of “*1/b*” for CMgS was higher than CMgS with drugs. The lower value of “*1/b*” of CMgS in the presence of drugs is indicative of the reduction in cohesive forces. In other words, the drugs increased the total degree of plastic deformation of CMgS during the compression process. These results positively correlate with Heckel parameter “*Py*”.

### 2.4. Physical Properties of the Tablets

The physical properties of all the tablet formulations are presented in Table 3. All tablets passed the current USP friability test, showing the % friability value well below the 1% upper level of acceptability for pharmaceutical tablets (USP, 2007). The formulated tablets showed thicknesses that are comparable to their respective commercial products. Acceptable content uniformity of the produced tablets was obtained ranging from 98 ± 2 to 101 ± 2%. Regarding the crushing strength, the results are in good agreement with the friability data and are considered to be acceptable values (>40 N) [17]. The higher thickness values for tablets made using CMS compared to that of Avicel^®^ 200 tablets is due to the high elasticity of these material.

**Figure 4:**
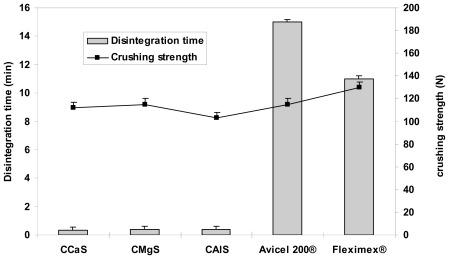
Crushing strength and disintegration times of tablets prepared from IBU and CCaS, or CMgS, or CAlS, or Avicel 200® excipients. Fleximex^®^ was used as a model commercial drug reference.

**Figure 5:**
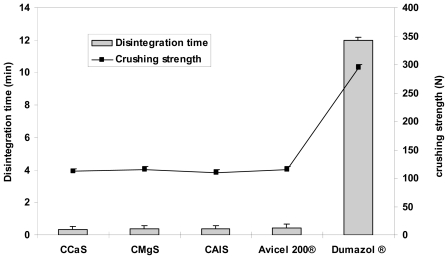
Crushing strength and disintegration times of tablets prepared from MET and CCaS, or CMgS, or CAlS, or Avicel 200® excipients. Dumazol^®^ was used as a model commercial drug reference.

**Figure 6:**
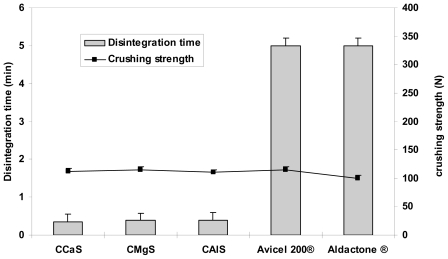
Crushing strength and disintegration times of tablets prepared from SPL and CCaS, or CMgS, or CAlS, or Avicel 200® excipients. Aldactone^®^ was used as a model commercial drug reference.

Figures 4–6 represent the crushing strengths and the disintegration times of the tablets prepared by direct compression using CMS in comparison with Avicel^®^ 200 (one of the most commercially used excipient in direct compression) and the respective commercial products. The crushing strengths of all tablet formulation were within 100–115 N. No significant difference (*p* > 0.05) in the crushing strengths was found between the three CMS. It showed superdisintegration properties. The three drug formulations disintegrated in less than one minute (*p* > 0.05). On the other hand, Avicel^®^ 200 tablets disintegrated in 15 min, less than one minute, and 5 min for IBU, MET, and SPL tablets, respectively.

The CMgS was used as intra-granular as well as extra-granular in the preparation of IBU and MET tablet formulations using the wet granulation method. The crushing strength and disintegration time results obtained are summarized in Table 4.

From the above results it can be concluded that intra-granular method resulted in harder tablets than extra-granular method, and this could be due to the binding effect of water used in the granulation. Intra-granulation enhances distribution of the inactive material (CMgS) in closer fashion around the active particles, enhancing bond formation between the active and inactive materials, which both form a single entity as a result of drying. The superdisintegration property of CMgS was not lost due to wet granulation; the tablets disintegrate in less than one minute. Such results enforce the idea that this multifunctional excipient can be used in dry and wet granulation with preferential addition as intragranulation method.

### 2.5. Drug Release Studies

**Figure 7:**
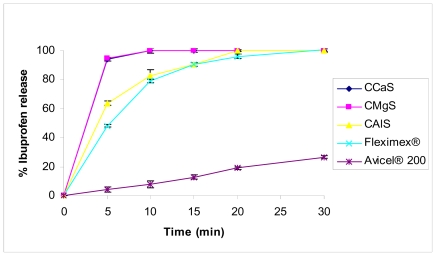
Dissolution profile of IBU formulated with CCaS, CMgS, CalS, and Avicel^®^ 200 release profile. Fleximex^®^ was used as a model commercial drug reference.

For IBU tablets, Figure 7, the CMgS and CCaS (Formulas F1 and F2, respectively) released 94% of the drug in 10 min while 84% of IBU was released from CAlS tablets (Formula F3) within the same time period. 100% of the drug was released from CMgS and CCaS tablets in 10 min, while 20 min and 30 min was required to release the same amount of the drug from CAlS and Fleximex^®^ tablets, respectively. This criterion of dissolution fulfills the USP criteria for immediate release solid dosage forms. Avicel^®^ 200, in contrast, released only 25% of the drug in 30 min. CalS, which is considered to be acidic filler, resulted in an acidic pH-microenvironment around the acidic IBU particles. This could be the reason behind the slower release profile compared to the basic fillers CcaS- and CMgS-containing tablets. In a similar study, [17] used cellulose II powder as a single filler excipient with multi functional action. Drug release from the tablets containing cellulose II powder was faster than both the commercial product (Advil^®^) and Avicel^®^ pH 102 containing tablets. They related this observation to the faster disintegration of cellulose II containing tablets. It seems that the use of a single excipient composed of chitin-Mg, Ca silicate mixtures can be used alone to yield an immediate release tablet of IBU similar to what has been reported.

The effect of the type of CMS was not significantly noticed on MET, since MET is highly soluble in the dissolution medium used. Complete MET release was within 5 min from all types of CMS. These results reveal that all tablet formulations met the USP drug release tolerance criterion.

**Figure 8:**
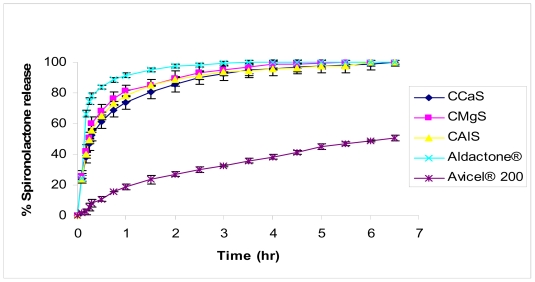
Dissolution profile of SPL formulated with CCaS, CMgS, CalS, and Avicel^®^ 200 release profile. Aldactone^®^ was used as a model commercial drug reference.

For SPL tablets, preliminary data using release studies using the USP conditions (1000 mL of 0.1 N HCl containing 0.1% sodium lauryl sulfate, Paddle, 75 rpm) doesn’t indicate any significant difference (*p* > 0.05) between the commercial product and CMS containing tablets. This could be attributed to the presence of hydrochloric acid as an acidic medium that could level off the micro-environmental pH differences between different metal silicates present in the CMS tablets. Consequently, water was used as a dissolution medium in order to get an insight about the differences in the microenvironment pH and its effect on drug release. The comparative release profiles of the water insoluble drug, SPL, are presented in Figure 8. The release rate decreased in the order: Aldactone^®^ > CMgS ~ CAlS ~ CCaS > Avicel^®^ 200. All tablet formulations made using CMS showed negligible difference in the dissolution profile (p < 0.05). Certainly, CMS type did not affect the dissolution profile of SPL, since it is a neutral drug. The CMS containing formulas showed a significant improvement in drug dissolution rate compared to Avicel^®^ 200 containing formula. However, the market formulation of SPL, Aldactone^®^, showed a faster dissolution rate than CMS made formula, this could be due to the many excipients included in Aldactone^®^ (such as calcium sulfate, corn starch, hypromellose, polyethylene glycol, povidone) [18,19]. SPL is water insoluble drug and to have a dissolution profile comparable with the leading brand is a real advance in excipient technology, particularly because its formulation is only containing the present multifunctional excipient.

### 2.6. Tablets Prepared by Wet Granulation

**Figure 9:**
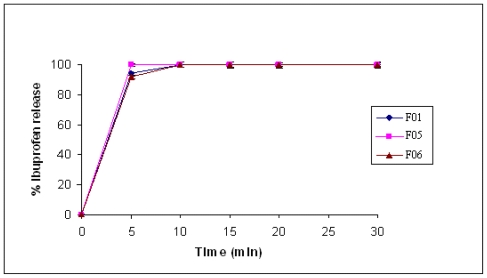
Dissolution profile of IBU formulated with CMgS by direct compression (F01), inter granulation (F05), and intra granulation (F06).

**Figure 10:**
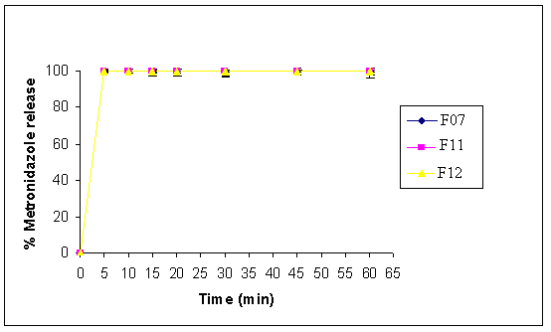
Dissolution profile of MET formulated with CMgS by direct compression (F07), inter granulation (F11), and intra granulation (F12).

As representative examples, CMgS with IBU and MET were selected to study the effect of wet granulation on the dissolution rate of tablets. Figures 9 and 10 illustrate that there was no significant difference in drug release between the wet granulation (either inter or intra) and direct compression using this novel multifunctional tablet excipient as a single tablet component. It is evident from Figures 9 and 10 that regardless of the method used for preparing tablets, the compendial dissolution of starting material is obtained. This shows that this excipient is suitable in both dry and wet granulation.

## 3. Experimental

### 3.1. Materials

Chitin (200 μm, Mwt of approximately 600 KD, JBICHEM, Shanghai, China), Microcrystalline cellulose (Avicel^®^ 200, FMC BioPolymer, Philadelphia, PA 19103, USA), Sodium silicate penta-hydrate (Na_2_SiO_3_.5H_2_O, BDH, Poole, England), Aluminum chloride hexa-hydrate (AlCl_3_.6H_2_O, MERCK KGaA, Darmstadt, Germany), Magnesium chloride hexa-hydrate (MgCl_2_.6H_2_O, SIGMA Chemical Co, St. Louis, MO 63178, USA), Calcium chloride di-hydrate (CaCl_2_.2H_2_O, ACROS Organics, Geel, Belgium), Ibuprofen (Boots Pharmaceuticals, UK), Metronidazole (G.D Seark and company, USA), Spironolactone (Micro, Novachem, India), all raw materials were donated by the Jordan Pharmaceutical Manufacturing Co. (JPM, Naor, Jordan). Hydrochloric acid solution conc. 37% (HCl, Scharlau Chemie S.A, Spain), Potassium di-hydrogen phosphate (KH_2_PO_4_, Merck KGaA, Germany), Sodium hydroxide pellets (NaOH, ACROS organics, USA). The reference tablets were as follows; Fleximex^®^ 400 mg tablets, Film coated (Alpharma Aps Company, Copenhagen, Denmark) as Ibuprofen tablets, Dumazol^®^ 500 mg tablets, Film coated (Alpharma Aps, Copenhagen, Denmark) as Metronidazole tablets, Aldactone^®^ 25 mg tablets, Film coated (SEARLE Company, High Wycombe, England) as Spironolactone tablets. All other reagents used were of analytical grade.

### 3.2. Methods

#### 3.2.1. Preparation of Three Chitin Metal Silicates (CMS): Chitin-Ca, -Mg and -Al Silicate Co Precipitates

All chitin-Ca, -Mg, and –Al silicate co precipitates were prepared according to the method described by Rashid *et al*, 2009 [12]. Briefly, into three separate solutions, each containing 10.0 g of sodium silicate dissolved in 400 mL deionized water, 7.5, 10, and 11.5 g of chitin was added. Respectively, 6.0 g of AlCl_3_.6H_2_O, 9.6 g of MgCl_3_.6H_2_O, 6.9 g of CaCl_3_.2H_2_O were added stoichiometrically and allowed to react with sodium silicate to produce suspended particles of the insoluble metal silicate that co precipitated onto chitin. The particles were filtered out using 20–25 μm filter papers (ALBET 135, Quantitative, Spain) then washed with deionized water. The product was dried in the oven at 90 °C, and finally passed through a mesh of 425 μm size and kept for further testing and characterization.

#### 3.2.2. Characterization of the Compacts

##### 3.2.2.1. Compatibility Study

For DSC studies, about 5 ± 0.5 mg of each of the drugs (IBU, MET and SPL), the three CMS (CCaS, CMgS and CAlS) and a mixture (1:1 drug to CMS w/w) were weighed into an aluminum crucible, which was then pierced and the DSC run was carried out using DSC 823, Mettler Toledo Star system, Switzerland. The instrument was initially calibrated with indium. Scanning runs were performed under atmospheric conditions. A heating rate of 10 °C/min was used for all samples.

##### 3.2.2.2. Characterization of Compacts by Heckel and Kawkita Plots

Chitin- metal silicate (Al, Mg, Ca), Ibuprofen (IBU) and spironolactone (SPL) were separately compressed by direct compression using a universal testing machine (UTM, RKM 50, PR-F System; ABS Instruments Pvt., Ltd., Leipzig, Germany). This was achieved without lubrication of the upper and lower punches as well as the dye. The punch speed was fixed at 10 mm/min. Different compression forces from 2.7–44.2 MPa were applied. Three tablets were prepared to ensure reproducibility. The tablets were flat, round with a 12 mm diameter, and 400 mg in weight. The compression behavior of the prepared compacts was evaluated according to Heckel and Kawakita equations.

The compression behavior of the samples was evaluated using the Heckel equation (Equation 4)

(4) $$\text{ln}\left(\frac{1}{1-D}\right)=KP+A$$

where D is the relative density of the compact at pressure *P; K* is the slope; and *A* is the intercept of the straight line obtained by linear regression from the Heckel plot.

The relative density, *D_0_*, of the powder at the point when the applied pressure equals zero is used to describe the initial rearrangement phase of densification as a result of die filling and high value indicating very dense packing. The relative densities *D_A_* and *D_0_* were calculated from Equations (5) and (6), respectively:

(5) $$D_{A}=1-{\text{e}}^{-\text{A}}$$

(6) $$D_{0}=1-{\text{e}}^{-{\text{A}}_{0}}$$

where *A_0_* represents the intercept of the line at *P*=0. The difference between *D_A_* and *D_0_* represents the extent of particle rearrangement (*D_B_*). The relative density *D_B_*, describes the phase of arrangement during the initial stages of compression. The extent of this depends on the theoretical point of densification at which particle deformation begins. The mean yield pressure (*P_y_*) was obtained as the reciprocal of the slope of the linear section in the curve. *Py* is inversely related to the ability of the material to deform plastically under pressure [20,21].

The Kawakita equation (Equation 7) is used to study powder compression via the degree of volume reduction, C. The basis for the Kawakita equation for powder compression is that particles subjected to a compressive load in a confined space are viewed as a system in equilibrium at all stages of compression, so that the product of the pressure term and the volume term is a constant [22]. Equation (1) above can be rearranged in linear form as:

(7) $$\frac{P}{C}=\frac{P}{a}+\frac{1}{ab}$$

Where, C is the degree of volume reduction of powder column under the applied pressure, P. The constant *a* is the minimum porosity of the material before compression while the constant *b* relates to the plasticity of the material. The reciprocal of *b* defines the pressure required to reduce the powder bed by 50%.

The degree of particle rearrangement can be affected simultaneously by the two Kawakita parameters, *a* and *b*. The combination of these into a single value, *i.e.,* the product of the Kawakita parameters *a* and *b*, may hence be used as an indicator of the degree of particle rearrangement during compression.

#### 3.2.3. Tablet Preparation

##### 3.2.3.1. Preparation of Tablets by Direct Compression Method

The composition of tablet formulations is presented in Table 5. The amounts per tablet of each of the three chitin-metal (Ca, Mg, and Al) silicate powders (particle size of 425 μm) or Avicel^®^ 200 and the drugs: ibuprofen (IBU), metronidazole (MET) or spironolactone (SPL) were separately and accurately weighed and mixed. The pre-weighed mixture was introducing manually in 12 mm die and compressed using a hydraulic press (C-30 Research and Industrial Instruments, London). The compression forces were set to produce tablets of nearly similar crushing strength and were within 10 and 150 kN. For each type of drug, a minimum of 100 tablets were prepared.

##### 3.2.3.2. Preparation of Tablets by Wet Granulation Using Chitin-Mg Silicate

For wet intra-granulation; CMgS powder and IBU or MET were sieved and blended in a cube mixer for 15 min. The mixture was then transferred to a planetary mixer and sufficient water was added to form a coherent wet mass. The wet mass was passed through a 2.50 mm sieve and then dried in a hot air oven at 50 °C for enough time to reach a moisture content of <2%. Moisture content was analyzed by an Ultra X moisture analyzer (August Gronert Co., Germany). The dried granules were further passed through a 0.80 mm sieve. Suitable weight of the final mixture equivalent to 400 mg and 500 mg of IBU and MET, respectively, was manually filled in the die and compressed to similar crushing strength using the hydraulic press.

For wet extra-granulation; IBU and MET were granulated separately to the end point with distilled water, the wet mass was passed over 2.50 mm sieve, then dried in a hot air oven at 50 °C for enough time to reach a moisture content of <2%, then the dried granules were re-sieved using 0.80 mm sieve. CMgS powder was mixed using the cube mixer with the dried granules of the active ingredients. Suitable weight of the final mixture equivalent to 400 mg and 500 mg of IBU and MET, respectively, was manually filled in the die and compressed to similar crushing strength using the hydraulic press. The amounts of drugs excipient and process used are shown in Table 5.

#### 3.2.4. Tablet Characterization

##### 3.2.4.1. Tablet Thickness

The thickness of 10 randomly selected tablets was determined using a vernier caliper (For-bro Engineers, Mumbai, India). The results are expressed as mean values of 10 determinations.

##### 3.2.4.2. Drug Content Uniformity

Ten tablets of each drug formulation were weighed individually, crushed and the drug was extracted in water. The solution was filtered through a cellulose acetate membrane (0.45 μm) and the drug content was determined by UV spectroscopy ((DU^®^ 6401, BECKMAN COULTER, USA) after a suitable dilution.

##### 3.2.4.3. Dissolution Test

The dissolution tests were performed using (Erweka DT6, Germany) dissolution apparatus and the test conditions are presented in Table 6. The amount of drug released was analyzed spectrophotometrically by measuring the absorbance using UV spectrophotometer (DU^®^ 640i, BECKMAN COULTER, USA) at λ 222 nm, λ 275 and λ 242 nm for IBU, MET and SPL, respectively. The amounts released were quantitated by the calibration curve method: the concentration of the standard solutions used ranged from 0.004 to 0.025 mg/mL, from 0.004 to 0.024 mg/mL and from 0.005 to 0.014 mg/mL for IBU, MET and SPL, respectively.

### 3.3. Statistical Analysis

Statistical analysis was carried out to compare the effect between different groups or parameters using the Analysis of Variance (ANOVA) on a computer software GraphPad Prism ® 4 (GraphPad Software Inc. San Diego, USA). Tukey-Kramer multiple comparison tests was used to compare the individual differences between the starches. At 95% confidence interval, *p* values less than or equal to 0.05 were considered significant.

## 4. Conclusions

The novel CMS co-precipitates could be used as a single filler tablet excipient with multifunctional action. The shortcoming excipients such as high moisture and loss of compressibility by MCC upon wet granulation were overcome by such a filler. They were successfully used to prepare tablets by direct compression, which is the process of choice for tablet manufacturing. Tablets produced using these fillers showed superiority regarding disintegration and dissolution profiles when compared with commercial products and with those prepared using Avicel^®^ 200. Tablets produced using these fillers also showed good crushing strength and met the USP friability tolerance limit. These fillers showed compatibility with acidic drug (ibuprofen), basic drug (metronidazole) as well as neutral drug (spironolactone). The compression behavior of the produced tablets represents B-type material, *i.e.*, initial phase of fragmentation followed by plastic deformation. The onset of plastic deformation of CMgS (as a representative example of CMS) increased in the presence of drugs. This could be useful in avoiding problems of capping and lamination in high speed tabletting machines.

## Figures and Tables

**Table 1:** Parameters obtained from Heckel plots.

Formula	*P_y_*	A	*D_A_*	*A_0_*	*D_0_*	*D_B_*
IBU(400 mg) + CMgS(300 mg)	81.97	1.7	0.82	0.4811	0.38	0.44
MET(500 mg) + CMgS(300 mg)	86.21	1.53	0.78	0.3592	0.30	0.48
SPL(25 mg) + CMgS(275 mg)	103.10	1.19	0.70	0.2324	0.21	0.49
Chitin Mg-silicate	153.85	0.72	0.51	0.1641	0.15	0.36

**Table 2:** Parameters obtained from Kawakita plots.

Formula	*1/ab*	*1/a*	*a*	*1/b*	*R^2^*
IBU + CMgS	0.33	1.16	0.86	0.29	1.0000
MET + CMgS	0.80	1.24	0.80	0.36	0.9998
SPL + CMgS	0.49	1.15	0.87	0.43	1.0000
CMgS	0.82	1.17	0.85	0.69	1.0000

**Table 3:** Physical properties of the prepared tablets.

Formula #	Drug	Tablet content (%)	Tablet thickness (mm)	Tablet friability (%)
F1	IBU	98 ± 2	0.71 ± 0.04	0.32 ± 0.05
F2	IBU	101 ± 2	0.73 ± 0.05	0.42 ± 0.05
F3	IBU	98 ± 03	0.74 ± 0.05	0.31 ± 0.05
F4	IBU	100 ± 2	0.63 ± 0.04	0.40 ± 0.03
F5	IBU	99 ± 2	0.74 ± 0.04	0.36 ± 0.05
F6	IBU	101 ± 2	0.75 ± 0.04	0.33 ± 0.04

F7	MET	98 ± 2	0.63 ± 0.04	0.29 ± 0.05
F8	MET	99 ± 2	0.65 ± 0.04	0.38 ± 0.03
F9	MET	98 ± 2	0.62 ± 0.04	0.35 ± 0.04
F10	MET	101 ± 2	0.57 ± 0.04	0.36 ± 0.05
F11	MET	99 ± 2	0.63 ± 0.04	0.42 ± 0.03
F12	MET	101 ± 2	0.63 ± 0.04	0.39 ± 0.04

F13	SPL	100 ± 2	0.38 ± 0.04	0.41 ± 0.05
F14	SPL	98 ± 2	0.39 ± 0.04	0.44 ± 0.05
F15	SPL	99 ± 2	0.40 ± 0.03	0.39 ± 0.05
F16	SPL	100 ± 2	0.29 ± 0.05	0.47 ± 0.05

**Flexamex®**	IBU	101 ± 2	0.57 ± 0.05	NR*
**Dumozole®**	MET	99 ± 2	0.63 ± 0.05	NR
**Aldactone®**	SPL	98 ± 2	0.36 ± 0.04	0.56 ± 0.05

* NR means not required due to the presence of a film coat.

**Table 4:** Effect of wet granulation on crushing strength and disintegration time of CMgS tablets. WIG: wet intragranulation, WEG: wet extragranulation.

Formula #	Crushing strength (N)	Disintegration time (min)
F05 (WIG, IBU)	104 ± 5	<1 min
F06 (WEG, IBU)	75 ± 5	<1 min
F11 (WIG, MET)	71 ± 5	<1 min
F12 (WEG, MET)	43 ± 5	<1 min

**Table 5:** Composition of model ibuprofen, metronidazole and spiranolactone tablet formulations.

Formula #	Drug*	Process	CMgS*	CCaS*	CALS*	Avicel® 200*
F 1	400 (IBU)	DC	300			
F 2	400 (IBU)	DC		300		
F 3	400 (IBU)	DC			300	
F 4	400 (IBU)	DC				300
F 5	400 (IBU)	WIG	300			

F 6	400 (IBU)	WEG	300			
F 7	500 (MET)	DC	300			
F 8	500 (MET)	DC		300		
F 9	500 (MET)	DC			300	
F 10	500 (MET)	DC				300
F 11	500 (MET)	WIG	200			

F 12	500 (MET)	WEG	200			
F 13	25 (SPL)	DC	275			
F 14	25 (SPL)	DC		275		
F 15	25 (SPL)	DC			275	
F 16	25 (SPL)	DC				27

DC: direct compression, WIG: wet intragranulation, WEG: wet extragranulation.

* Amounts in milligrams.

**Table 6:** Dissolution conditions used for IBU, MET and SPL tablets.

Ingredient	IBU tablet	SPL tablet	MET Tablet
USP Apparatus	II	II	II
Dissolution medium (900 mL)	phosphate buffer pH7.2	Water	0.1 N HCl
Agitation rate (rpm)	50	75	100
Temperature (°C)	37	37	37

## References

[1] Silverstein I Excipient GMP Quality Standards One Is Enough Pharm Technol 2002 25 46 52

[2] Armstrong NA Functionality Related Tests for Excipients Int J Pharm 1997 155 1 5

[3] Van de Waterbeemd H Testa B Drug Bioavailability: Estimation of Solubility, Permeability, Absorption and Bioavailability 2 Wiley-VCH New York, NY, USA 2008

[4] Jackson K Young D Pant S Drug-excipient interactions and their affect on absorption Pharm Sci Technol Today 2000 3 336 345 11050458 10.1016/s1461-5347(00)00301-1

[5] Monedero Perales MC Munoz-Ruiz A Velasco Antequera MV Munoz Munoz N Jimenez-Castellanos MR Comparative Tableting and Microstructural Properties of a New Starch for Direct Compression Drug Develop Ind Pharm 1996 22 689 695

[6] Hwang R Peck GR A Systematic Evaluation of the Compression and Tablets Characteristics of Various Types of Microcrystalline Cellulose Pharm Technol 2001 24 112 132

[7] Reimerdes D Aufmuth KP Tabletting with Co-processed Lactose-Cellulose Excipients Manuf Chem 1992 63 21 24

[8] Lourdes Garzo’n M Villafuerte L Compactibility of mixtures of calcium carbonate and microcrystalline cellulose Int J Pharm 2002 231 33 41 11719011 10.1016/s0378-5173(01)00857-2

[9] Patel V Patel M Patel R Chitosan: A Unique Pharmaceutical Excipient Drug Deliv Technol 2005 6 30 40

[10] El-Barghouthi M Eftaiha A Rashid I Al-Remawi M Badwan A A Novel Superdisintegrating Agent Made from Physically Modified Chitosan with Silicon Dioxide Drug Develop Ind Pharm 2008 34 373 383 10.1080/03639040701657792 18401779

[11] Rashid I Al-Remawi M Eftaiha A Badwan A Chitin silicone dioxide coprecipitate as a novel superdisintegrant J Pharm Sci 2008 97 4955 4969 18314884 10.1002/jps.21354

[12] Rashid I Daraghmeh N Al-Remawi M Leharne S Chowdhry B Badwan A Characterization of Chitin-Metal Silicates as Binding Superdisintegrants J Pharm Sci 2009 98 4887 4901 19691098 10.1002/jps.21781

[13] Mura P Manderioli A Bramanti G Furlanetto S Pinzauti S Utilization of differential scanning calorimetry as a screening technique to determine the compatibility of ketoprofen with excipients Int J Pharm 1995 119 71 79

[14] Verma R Garg S Selection of excipients for extended release formulations of glipizide through drug–excipient compatibility testing J Pharmceut Biomed 2005 38 633 644 10.1016/j.jpba.2005.02.026 15967291

[15] Martins E Christiana I Olobayo K Effect of *Grewia* gum on the mechanical properties of Paracetamol tablet formulations Afr J Pharm Pharmaco 2008 2 001 006

[16] Odeku OA Patani BO Evaluation of dika nut mucilage (Irvingia gabonensis) as binding agent in metronidazole tablet formulations Pharm Develop Technol 2005 10 439 446 10.1081/pdt-54477 16176024

[17] Gohel MC Parikh RK Brahmbhatt BK Shah AR Improving the tablet characteristics and Dissolution Profile of Ibuprofen by Using a Novel Coprocessed Superdisintegrant AAPS PharmSciTech 2007 8 Article 13 10.1208/pt0801013 PMC2750448 17408213

[18] Medina MR Kumar V Evaluation of cellulose II powders as a potential multifunctional excipient in tablet formulations Int J Pharm 2006 322 31 35 16828996 10.1016/j.ijpharm.2006.05.033

[19] RxList The Internet Drug Index Aldactone^®^, (spironolactone) Tablets, USP 2008 1 8

[20] Paronen P Iilla J Porosity-Pressure Functions Pharmaceutical Powder Compaction Technology Marcel Dekker Inc New York, NY, USA 1996 55 75

[21] Itiola OA Compressional characteristics of three starches and the mechanical properties of their tablets Pharm World J 1991 8 91 94 10.1081/ddc-120003857 12149958

[22] Kawakita K Ludde KH Some considerations on powder compression equations Powder Technol 1971 4 61 68

